# Evaluation of Bioequivalence of Two Long-Acting 20% Oxytetracycline Formulations in Pigs

**DOI:** 10.3389/fvets.2017.00061

**Published:** 2017-05-01

**Authors:** Zhixin Lei, Qianying Liu, Bing Yang, Saeed Ahmed, Jincheng Xiong, Tingting Song, Pin Chen, Jiyue Cao, Qigai He

**Affiliations:** ^1^National Reference Laboratory of Veterinary Drug Residues, MAO Key Laboratory for Detection of Veterinary Drug Residues, Huazhong Agriculture University, Wuhan, China; ^2^State Key Laboratory of Agriculture Microbiology, College of Veterinary Medicine, Huazhong Agriculture University, Wuhan, China

**Keywords:** oxytetracycline, pharmacokinetics, bioequivalence, formulations, confidence interval

## Abstract

The aim of this study was to explore the bioequivalence of long-acting oxytetracycline in two formulations, a reference formulation (Terramycin 20% LA, Pfizer) and a test one (Kangtekang 20% LA, Huishen). Both formulations were administered intramuscularly at 20 mg/kg body weight at each of 24 healthy animals during a two-period crossover parallel experimental design. The oxytetracycline (OTC) concentrations in plasma were measured by high-performance liquid chromatography, and the limit of quantification was 0.05 µg/ml with a recovery ratio of above 90%. Moreover, the descriptive pharmacokinetics parameters (*C*_max_, AUC_0–144h_, and AUC_0–∞_) were calculated and compared under analysis of variance, and 90% confidence interval (CI) were compared, except for *T*_max_ analyzed by non-parametric tests based on Wilcoxons’s signed rank test. The comparison results of *C*_max_, AUC_0–144h_, AUC_0–∞_, and *T*_max_ were 5.066 ± 0.486, 5.071 ± 0.877 µg/ml, 118.926 ± 13.259, 126.179 ± 17.390 µg h/ml, 123.087 ± 13.906, 130.732 ± 18.562 µg h/ml, 0.740 ± 0.278, 0.650 ± 0.258 h, respectively, and did not reveal any significant differences. In addition, 90% CIs of these ratios for reference and test product were within an interval of 80–125%, and the relative bioavailability of test one was (94.291 ± 15.287)%. Therefore, it has been concluded that test OTC was bioequivalent to the reference formulation in pigs.

## Introduction

Oxytetracycline (OTC), a broad-spectrum antibiotic, is widely used to treat Gram-positive (*Streptococcus* sp., *Staphylococcus* sp.) and Gram-negative (*Escherichia coli*) infections in animals (1–3). OTC is a tetracycline derivative produced by *Streptomyces rimosus* (4, 5). Long-acting oxytetracycline (LOTC) formulations are used to treat disease by maintaining an effective concentration in animals for 2 or 3 days. It is manufactured easily and used widely in developing countries as veterinary medicine (6). Long-acting injection can be performed to increase OTC and keep high concentrations in plasma above minimum inhibitory concentrations for several days, decreasing the number of administration per treatment (7, 8). As first LOTC receiving a market authorization, Terramycin LA (Pfizer) is the reference formulation for bioequivalence studies (6, 9–11).

Bioequivalence studies play an important role in new animal drug implementation and to support supplemental application in case of modification of dosage forms, administration routes, or manufacturing process that may have a significant effect on bioavailability (12, 13). Bioequivalence guidelines are established by respective organizations, while the most well-known guidelines are those of Europe and America. As a guideline to bioequivalence in the US, two products are considered for bioequivalence studies, when they are as follow bioequivalent, those in therapeutic ingredient or active ingredients, and available at the site of drug action, affected drug in assimilation rate and extent (14–18). In addition, a guideline in Europe states that bioequivalence studies should be performed within acceptable limits for the active ingredients between two products or administration routes under appropriate conditions (19–21).

According to the European medicines agency guidelines, a pharmacokinetic parameters comparison between two formulations is the best way for a bioequivalence examination of veterinary drugs, in which the area under the plasma concentration time curve to last concentration (AUC_0–t_), the area under the plasma concentration time curve extrapolated to infinity (AUC_0–∞_), the peak maximum plasma concentration (*C*_max_), and the time to maximum concentration (*T*_max_) are used for bioequivalent analysis (21). Once the bioequivalence has been demonstrated between two formulations, the clinical efficacy of test formulation is equivalent to those observed during the clinical trials of the reference formulation (22–24).

For OTC bioequivalence and pharmacokinetics studies, most investigations have been conducted in cattle and chickens, but fewer in pigs (6, 10, 25–28).

## Materials and Methods

### Drugs and Reagents

Oxytetracycline standard (96.5%), lot number: 15820000, was used for calibration of the analytical method. Two kinds of commercial products of OTC injection containing 20% OTC—a test one (Kangtekang 20% LA, Huishen, lot number: 10453036AA, produced from China) and a reference one (Terramycin 20% LA, Pfizer, lot number: 20050802, produced from America)—were selected for bioequivalence study. The other reagent used in this study was analar, purchased from Sinopharm Group Shanghai Chemical Reagent Co., Ltd.

### Animals

Twenty-four landrace healthy pigs (12 males, 12 females), 6 weeks old and weighing 25–30 kg were purchased from the Livestock and Poultry Breeding Center of Hubei Province (Wuhan, China). Pigs were raised with water and antibiotic-free feed to acclimatize for 1 week prior to first drug administration. The environment temperature and relative humidity of the pig enclosure were kept at 20–26°C and 45–65%, respectively.

### Bioequivalence Study Design and Sample Collection

The bioequivalence study was performed according to a crossover design with a washout period of 8 days. Pigs were divided randomly into groups A and B of 12 pigs by groups. Pigs in group A received the test product (Kangtekang 20% LA, Huishen) in the first period and the reference product (Terramycin 20% LA, Pfizer) in the second period and group B received drugs in the opposite order. The two products were administered intramuscularly at a dose of 20 mg/kg body weight. All animals were bred with water and antibiotic-free feed.

Animals were sampled during the following 6 days after i.m. administrating OTC products during each period. Plasma samples (3 ml) were collected at 0.083, 0.25, 0.5, 1, 2, 3, 6, 8, 12, 24, 36, 48, 72, 96, 120, and 144 h into tubes containing heparin after i.m. administration at a single dose of 20 mg/kg body weight. Samples were centrifuged at 3,000 rpm/s for 10 min, and then blood serum was obtained. Samples were stored at −20°C until analyzed by high-performance liquid chromatography (HPLC).

### HPLC Determination of OTC Concentration

A C18 reverse-phase column (250 mm × 4.6 mm, 5 µm i.d.; Agilent, USA) was used for HPLC, which was performed with a 355-nm detection wavelength at 30°C. Phases A, B, and C were 0.01 mol/l oxalic acid, acetonitrile, and methanol, respectively, which constituted the mobile phase of HPLC with a volume ratio (A:B:C, 83:10:7) for gradient elution. Plasma samples of 0.5 ml were mixed with 100 µl 10% perchloric acid with vortex oscillation at 3,000 rpm/s, then extracted to obtain supernatant liquid. Supernatants were evaporated to dryness under nitrogen at 45°C, followed by resuspension in the mobile phase at the initial volume, and then 20 µl of resuspension was injected directly into the HPLC system with 0.22 µm organic membranes.

### Pharmacokinetic and Bioequivalence Analysis

Descriptive PK parameters were obtained with WinNonlin Professional software (Version 5.2.1) (Certara, USA). The maximum plasma concentrations (*C*_max_) and time to *C*_max_ (*T*_max_) were obtained from the plasma concentration versus time data. AUC were calculated by the linear trapezoidal rule until the last sampling time (AUC_0–144h_) and with extrapolation to infinity (AUC_0–∞_). T_1/2_ was calculated from the terminal slope (β) estimated by log-linear regression according VICH guideline.

Analysis of variance (ANOVA) was used to compare and assess the effect of the formulations on the test formulation compared with the classical ones, with logarithmically transformed parameters of AUC_0–144h_, AUC_0–∞_ (Bioequivalence, August 2015) as recommended by technical guidelines for veterinary drug in China (29, 30). However, *T*_max_ comparison was performed with a Wilcoxon signed rank test. Parametric 90% confidence intervals (CIs) of the mean of test/reference ratios of AUC_0–144h_, AUC_0–∞_, and *C*_max_ were calculated using the residual variance of ANOVA with the assumption of a multiplicative model. CIs were calculated with SPSS analysis (IBM, USA).

### Statistical Analysis

The *p* values of <0.05 and <0.01 were considered statistically significant and extremely significant, respectively (*p < 0.05 and **p < 0.01).

## Results

### OTC HPLC Analysis in Plasma

**Figure 1:**
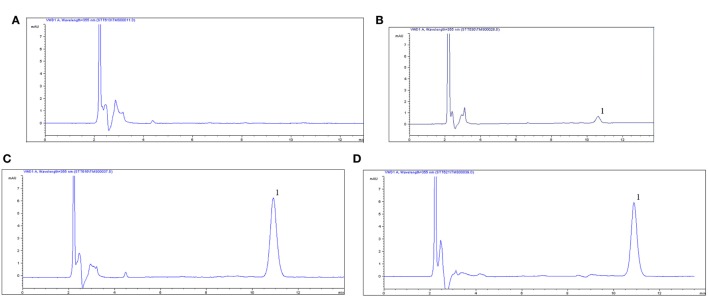
**Chromatograms of oxytetracycline (OTC) in plasma**. **(A)** Control blank group in plasma, **(B)** lower limit of quantification (0.05 µg/ml) of OTC in plasma, **(C)** chromatograms of plasma at 1 h after i.m. 20% OTC reference injection, **(D)** chromatograms of plasma at 1 h after i.m. 20% OTC test one, *1*, the peak time of OTC.

The proposed method was suitable for OTC quantification in plasma, showing good selectivity above 89% recovery and a good linear relationship from 0.05 to 10 µg/ml. The chromatogram in Figures 1A–D shows the blank, the lower limit of quantification (LLOQ), and measured samples at 5 min after i.m. of two formulation products in plasma, which indicate the proposed method for OTC detection are specific and accurate. The typical regression equation was *y* = 0.0507*A* − 0.0238, *R*^2^ = 0.9999. The LLOQ was 0.05 µg/ml in plasma presented in the Table 1. The coefficient of determination (*R*^2^) of standard curves from 0.05 to 10 µg/ml was 0.9999, and the inter-day and intra-day coefficient variation were below 5% in plasma. In addition, the recovery ratios were in the range of 89.13 ± 2.54 to 92.19 ± 3.75% in plasma (Table 2).

**Table 1:** **Sensibility of oxytertracycline in plasma**.

Samples	LLOD (μg/ml)	Lower limit of quantification (μg/ml)
Plasma	0.025	0.05

**Table 2:** **The result (mean ± SD) of method validation of oxytertracycline in plasma**.

Concentration (μg/ml)	Recovery (%)	Inter-day CV (%)	Intra-day CV (%)	Accuracy (RE%)
0.1	92.19 ± 3.75	3.17 ± 0.75	3.61 ± 0.65	7.96
1.0	90.80 ± 2.40	2.04 ± 0.76	4.07 ± 0.86	9.23
10	89.13 ± 2.54	2.08 ± 0.77	2.43 ± 0.78	10.86

*CV, coefficient of variability, RE, the accuracy*.

### Pharmacokinetic Analysis

**Figure 2:**
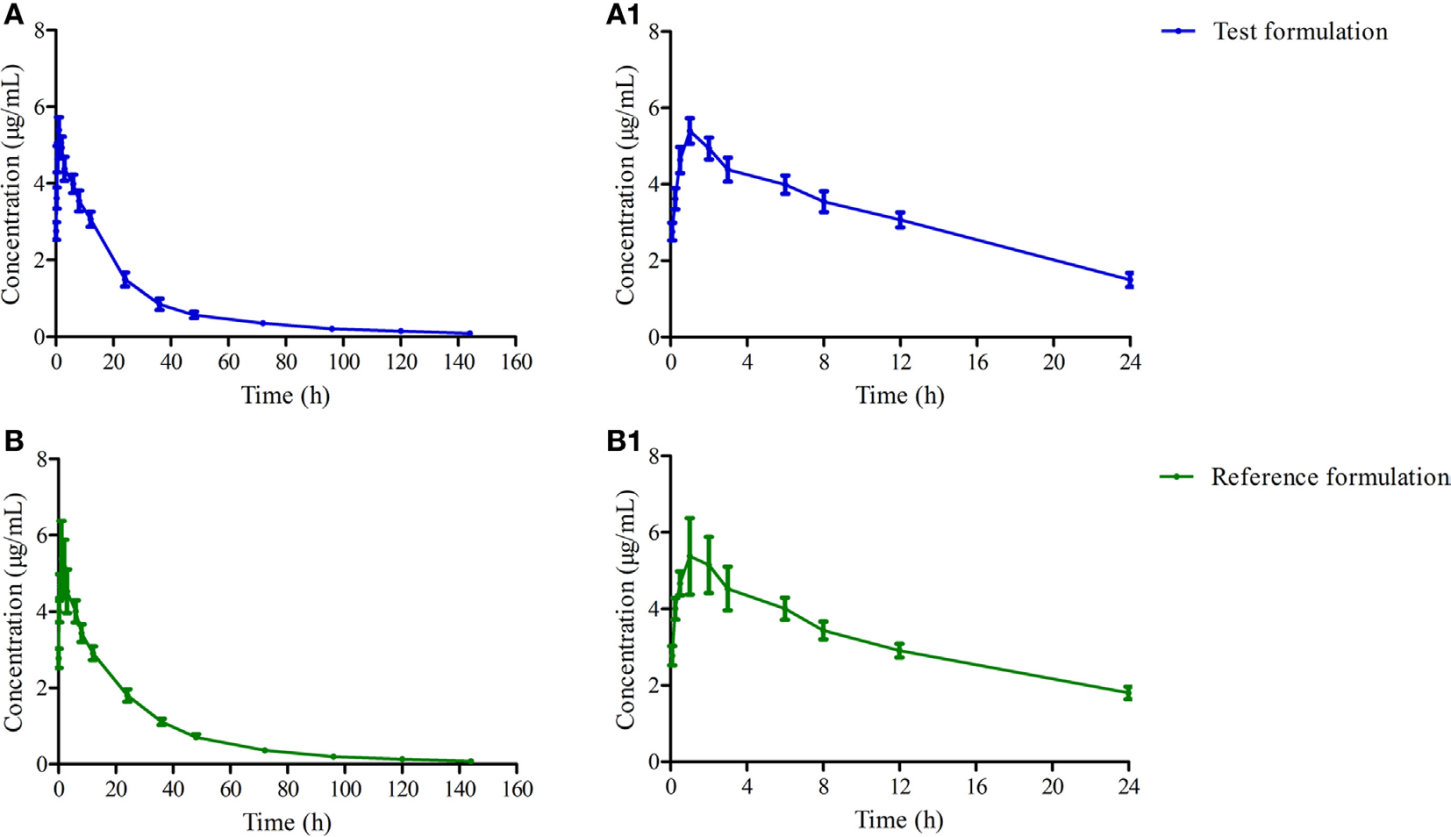
**The mean concentration time curves of test and reference formulations in plasma after i.m. (A) present the test one, (A_1_) present amplification of panel (A) at 0–24 h, (B) present the reference one, (B_1_) present amplification of panel (B) at 0–24 h**.

The mean ± SD of OTC concentration time profile are presented in Figure 2 after i.m. of two formulations, and the main descriptive PK parameters are reported in Table 3.

**Table 3:** **Pharmacokinetic parameters for the test formulation and reference formulation, *p* value and relative fraction**.

Parameters	Unit	Test	Reference	Analysis of variance	*F* (%)
AUC_0–144h_	μg h/ml	118.976 ± 13.259	126.179 ± 17.390	0.976	
AUC_0–∞_	μg h/ml	123.087 ± 13.906	130.732 ± 18.562	0.313	94.291 ± 15.287
*C*_max_	μg/ml	5.066 ± 0.486	5.071 ± 0.877	0.874	
*T*_max_	h	0.740 ± 0.278	0.650 ± 0.258	>0.05^a^	

*F represents relative bioavailability*.

*^a^Wilcoxon test*.

### Bioequivalence Analysis

Log-transformed *C*_max_, AUC_0–144h_, AUC_0–∞_ and untransformed *T*_max_ of the test formulation (Kangtekang 20% LA, Huishen) were compared with the reference one (Terramycin 20% LA, Pfizer) for a bioavailability study with ANOVA analysis and 90% CI. No statistically significant differences were observed for *C*_max_, AUC_0–144h_, AUC_0–∞_ in Table 3. The relative bioavailability of the test product compared to the reference one was 94.291 ± 15.287% (Table 3).

The two one-sided *T*-tests and 90% CI analysis displayed the ratios mean of log-transformed *C*_max_, AUC_0–144h_, AUC_0–∞_, and 90% CI on test to reference formulations were 99.5, 98.8, 99.1% and 91.9–107.2, 99.6–100.7, 92.0–102.1%, which were all in the range of 75–143% within the bioequivalence acceptance range (Table 4).

**Table 4:** **Two one-sided *T*-test and 90% of confidence interval (CI)**.

Parameters	*t*_1_	*t*_2_	90% CI	Ratio (*T*/*R*) (%)	Acceptable range (%)
AUC_0–144_	4.928*	9.812	99.6–100.7	98.8	80–125
AUC_0–∞_	6.335*	8.405	92.0–102.1	99.1	80–125
*C*_max_	11.486*	12.108	91.9–107.2	99.5	75–143

*Presents significant difference p < 0.05.

## Discussion

As long-acting OTC belongs to the tetracycline group, it is a broad-spectrum antimicrobial agent, presenting persistent actions and sustaining absorption from the depot site after i.m. injection (26). Most studies have reported PK and bioequivalence studies in cattle, sheep, goats, and rabbits, but only a few have investigated bioequivalence in pigs (6, 11, 31, 32). In the current study, the analytical methods for OTC quantification detection in plasma were specific, sensitive, accurate and were performed for bioequivalence study between the test product and the reference one in pigs. LOQ (0.05 µg/ml) was available for OTC detection in plasma, which was similar to the one previously described (0.05 µg/ml) by Nora Mestorino et al. and Brentnall et al. (6, 27).

AUC was a useful index for biological availability of the active moiety of a drug formulation regarding the absorption extent. Both AUC_0–144h_ and AUC_0–∞_ were 118.976 ± 13.259, 126.179 ± 17.390 and 123.087 ± 13.906, 130.732 ± 18.562 μg h/ml for test and reference products, which are higher than previous reports in pigs (33), but the ANOVA analysis revealed these two formulations had no significant differences with *p* > 0.05 (Table 3). *C*_max_ of these two products were similar to other reports in pigs—about 5.066 ± 0.486 and 5.071 ± 0.877 μg/ml—which presented a high drug concentration in plasma (Table 3) (34–36). Both formulations displayed similar plasma profiles and demonstrated that they were absorbed progressively, reaching *C*_max_ approximately 0.75 h after administration, keeping 1/20 *C*_max_ concentration in plasma after 120 h. *T*_max_, *C*_max_, and AUC_0–∞_ were 2.01 h, 3.98 µg/ml, 74.87 µg h/ml, and 0.609 h, 4.956 µg/ml, 112.483 µg h/ml in the research of Jie Bao and Xue Ling at a dose of 20 mg/kg, respectively (36, 37). In this study, a lower peak is reached later in comparison with our results. It might be related to the OTC concentration in the drug product, which was 30% higher than the 20% in our test and reference product studied. These results suggest a slower absorption of OTC with 30% formulation in comparison with 20% ones. These parameters of OTC in this study were similar to a previous study (35). The relative bioavailability of the test product compared to the reference one was 94.291 ± 15.287% (Table 3), and was included in the range 0.8–1.25, which satisfied the requirements of the Food and Drug Administration (21).

For a bioequivalence study of these two OTC injection formulations, and to make a statistical comparison, AUC_0–last_ and AUC_0–∞_, *C*_max_, and *T*_max_ were selected. When these parameters were not significantly different between the test and reference product, they will be bioequivalent if the CI were comparable. This was demonstrated through the ANOVA of log-transformed value and the two one-sided *T*-tests and 90% CI. In our study, the three parameters revealed there were no significant differences between the test and reference products (*t*_1_ and *t*_2_) in AUC_0–last_ and AUC_0–∞_, *C*_max_, which were all above 1.717 and had significant differences (*p* < 0.05) with each other (Table 4). The AUC_0–144h_ and AUC_0–∞_, *C*_max_ results presenting 90% of CI were inside the CIs (75–143) set by the European Union. *T*_max_ presented no significant difference by non-parametric analysis (Table 4). Thus, these results demonstrated that the test OTC injection (Kangtekang 20% LA, Huishen) was bioequivalent to the reference one (Terramycin 20% LA, Pfizer).

## Ethics Statement

The animals that were used in this study were conducted according to relevant guidelines and regulations of Animal Care Center, Hubei Science and Technology Agency in China (SYXK 2013-0044). Animal housing care and experimental protocol were conducted according to the regulation of experimental animal usage in Hubei province of China, and the protocol was approved by the Ethics Committee of Huazhong Agricultural University.

## Author Contributions

Experiment designation: ZL, QL, JC, and QH; carrying out the experiment: ZL, BY, and JX; manuscript writing: ZL; and manuscript review and modification: SA, TS, and PC.

## Conflict of Interest Statement

The authors declare that the research was conducted in the absence of any commercial or financial relationships that could be construed as a potential conflict of interest.
